# The Influence of Nickel-Titanium (Ni-Ti) Rotary Instrument Systems on Debris and Smear Layer Formation in Endodontic Procedures: An In Vitro Scanning Electron Microscopy Study

**DOI:** 10.7759/cureus.54310

**Published:** 2024-02-16

**Authors:** Sheetal Mali, Amit Patil, Deepak Sharma, Himmat Jaiswal, Hrishikesh A Saoji, Anamika Sinha, Ramanpal Singh

**Affiliations:** 1 Department of Conservative Dentistry and Endodontics, Bharati Vidyapeeth (Deemed to be University) Dental College and Hospital, Navi Mumbai, IND; 2 Department of Oral Medicine and Radiology, New Horizon Dental College And Research Institute, Bilaspur, IND

**Keywords:** scanning electron microscopy, xp endo, twisted file, protaper, ni-ti rotary instruments, smear layer, debris, endodontics

## Abstract

Background

Successful endodontic treatment relies on the effective removal of debris and the prevention of smear layer formation within the root canals. The choice of nickel-titanium (Ni-Ti) rotary instrument systems can significantly impact these outcomes.

Aim

This study aims to evaluate and compare the debris and smear layer formation in root canals of extracted mandibular second premolar teeth following instrumentation with the ProTaper Universal (Dentsply Sirona, Charlotte, NC) (Group II), Twisted File (Kerr Endodontics, Gilbert, AZ) (Group III), and XP Endo (FKG Dentaire, La Chaux-de-Fonds, Switzerland) (Group IV) Ni-Ti rotary instrument systems.

Methods

In this in vitro study, 60 extracted mandibular second premolar teeth were randomly divided into four groups, each containing 15 teeth. Group I served as the control with no instrumentation. Groups II, III, and IV were instrumented with the ProTaper Universal rotary file, the Twisted File, and the XP Endo file systems, respectively. Debris and smear layer formation were evaluated through scanning electron microscopy (SEM), and photomicrographs were scored using a standardized index.

Results

Group II (ProTaper) exhibited the highest mean debris and smear layer scores, with values of 3.50 and 2.70, respectively. Group IV (XP Endo) demonstrated the least debris and smear layer formation, with mean scores of 2.65 and 2.08, respectively. Statistical analysis confirmed significant differences among the groups for both debris and smear layer formation.

Conclusion

The results highlight the practical importance of selecting appropriate Ni-Ti rotary instrument systems to minimize debris and smear layer formation during endodontic procedures. The XP Endo file system showed promise as a favorable choice in this regard, but further clinical research is needed to validate these findings.

## Introduction

Biofilms are communities of microorganisms that adhere to surfaces and develop intricate structures, often encased in a protective matrix. In the context of endodontics, these biofilms primarily develop within the root canal system. The microorganisms involved can include bacteria, fungi, and other microorganisms, and they are known to be responsible for various endodontic infections. Understanding biofilm formation is crucial because it represents the starting point for comprehending the complexities of endodontic pathology [1]. Biofilms provide a protective environment for microorganisms, enabling them to resist the host's immune responses and the effects of antimicrobial agents, which makes them particularly challenging to manage. Apical periodontitis is a chronic inflammatory condition that affects the tissues surrounding the apex (tip) of the tooth root. It is often a consequence of biofilm-induced infection within the root canal system. Unlike irreversible pulpitis, which primarily involves the pulp tissue within the tooth, apical periodontitis requires more complex management strategies due to its location and the involvement of periapical tissues. Given its chronic nature and the potential for persistent infections, apical periodontitis represents a critical area of focus in endodontic treatment [1,2].

Endodontic therapy, the cornerstone of modern dentistry, is dedicated to preserving the vitality of the teeth afflicted by pulp pathosis, infection, or irreversible pulpitis. Achieving successful endodontic outcomes necessitates thorough the cleaning, shaping, and disinfection of the root canal system, ultimately leading to the elimination of pathogens and their byproducts, such as debris and smear layers [2].

The concept of the "smear layer" and "debris" within root canals was introduced by McComb and Smith in 1975 [3]. Debris refers to organic and inorganic material, including pulp tissue remnants, dentin shavings, and bacteria, generated during root canal instrumentation [4]. In contrast, the smear layer is a thin layer of compacted dentin particles, pulp tissue, and microorganisms created during mechanical instrumentation, ultimately covering the root canal walls [5]. Both debris and smear layers may hinder the penetration of irrigants and sealers, limiting their effectiveness in disinfecting and obturating the root canal system [6].

For the majority of treatment failures, nonsurgical retreatment is the initial course of action. To avoid reinfection, this needs to be meticulously disinfected. It is widely acknowledged that the removal of prior root-filling materials as extensively as feasible is necessary for the optimum debridement of the canal space [7]. To optimize the cleanliness and successful treatment of root canals, numerous endodontic instrumentation techniques and technologies have been developed. Nickel-titanium (Ni-Ti) rotary instruments have revolutionized root canal preparation, offering enhanced flexibility, efficiency, and precision compared to traditional stainless-steel files [8]. These Ni-Ti rotary systems, including the ProTaper Universal (Dentsply Sirona, Charlotte, NC) (Group II), Twisted File (Kerr Endodontics, Gilbert, AZ) (Group III), and XP Endo (FKG Dentaire, La Chaux-de-Fonds, Switzerland) (Group IV), have gained popularity in clinical practice for their diverse designs and claimed benefits in root canal cleaning and shaping.

However, the comparative effectiveness of these Ni-Ti rotary instrument systems in minimizing debris and smear layer formation within root canals remains a subject of ongoing research and debate. While various studies have examined the performance of these systems individually, a few have directly compared their influence on debris and smear layer formation within a single investigation. In this study, we aim to address the comparative effectiveness of nickel-titanium (Ni-Ti) rotary instrument systems, including the ProTaper Universal (Group II), Twisted File (Group III), and XP Endo (Group IV), in minimizing debris and smear layer formation within root canals.

The objective of this in vitro scanning electron microscope (SEM) study is to evaluate and compare the debris and smear layer formation in the root canals of extracted mandibular second premolar teeth following instrumentation with the ProTaper Universal (Group II), Twisted File (Group III), and XP Endo (Group IV) Ni-Ti rotary instrument systems. This investigation aims to shed light on the potential benefits and limitations of these instrument systems in the context of endodontic treatment, with the ultimate goal of enhancing clinical decision-making and improving the quality of patient care.

## Materials and methods

Sample preparation

Following meticulous selection and grouping of the teeth, each specimen underwent a standardized procedure for splitting to facilitate SEM analysis. Longitudinal grooves were carefully created on the mesial and distal surfaces of the teeth, parallel to the root's longitudinal axis, using a diamond disk. These grooves served as guides for the subsequent splitting process and were essential for ensuring consistency and accuracy. The splitting itself was conducted with precision, employing a chisel and hammer with gentle strokes to avoid damaging the root canals. From each split specimen, a segment was chosen based on the quality of the splitting, ensuring that representative portions were selected for SEM examination. This meticulous approach to tooth splitting maintained the integrity of the root canals and provided suitable specimens for detailed analysis.

After splitting, the selected segments from each specimen were prepared for SEM analysis to examine debris and smear layer formation within the root canals. This preparation involved the fixation of the specimens onto SEM stubs using conductive adhesive tabs to ensure stability during imaging. Subsequently, the specimens were sputter-coated with a thin layer of conductive material, typically gold or palladium, to enhance surface conductivity and minimize charging effects during SEM imaging. Once prepared, the specimens were loaded into the SEM chamber for examination at high magnifications (×1500 and ×5000). Photomicrographs were systematically captured to document the distribution and characteristics of debris and smear layer formation within the root canals. This standardized preparation procedure ensured consistency and reliability in SEM analysis, enabling a comprehensive evaluation of the experimental outcomes across all groups.

Selection and grouping of the teeth

A total of 60 extracted mandibular second premolar teeth were collected, and meticulous examinations were performed to ensure their suitability for the study. The teeth that displayed caries, fractures, or visible defects were excluded from the study. To maintain a consistent sample size, all selected teeth were decoronated at the cementoenamel junction, leaving a standardized root length of 15 mm. The visual inspection of the apical foramina confirmed their patency, ensuring that the teeth were suitable for further experimentation. Randomization was applied to distribute the teeth into four groups, with each group consisting of 15 teeth. This random allocation minimized any potential bias in group selection and ensured that each group had a uniform distribution of tooth characteristics. Consent for the collection of extracted mandibular second premolar teeth was obtained from individuals who had provided their teeth for dental procedures, and these teeth were no longer needed for clinical purposes.

The four groups were defined as follows: Group I served as the control group, where no instrumentation was performed, and the teeth remained untreated. Group II underwent root canal instrumentation using the ProTaper Universal rotary file (F2). In Group III, the Twisted File system was employed, utilizing instruments with an International Organization for Standardization (ISO) size of 0.25 and a 6% taper. Group IV was prepared using the XP Endo file system with an ISO size of 0.25.

Instrumentation procedure

During the instrumentation process for all teeth in each group, a standardized volume of 5 mL of normal saline was employed as the irrigating agent. The normal saline was carefully delivered through a syringe equipped with a 30-gauge fine needle to ensure the effective irrigation of the root canals. To facilitate the process, longitudinal grooves running parallel to the root's longitudinal axis were created on the mesial and distal surfaces of each specimen. A diamond disk was employed for this purpose, ensuring it did not penetrate the root canals. Subsequently, these grooves were used as guides to carefully split the specimens into two halves, using a chisel and hammer with gentle strokes. From each split specimen, a segment was chosen based on the quality of the splitting.

SEM examination

**Figure 1:**
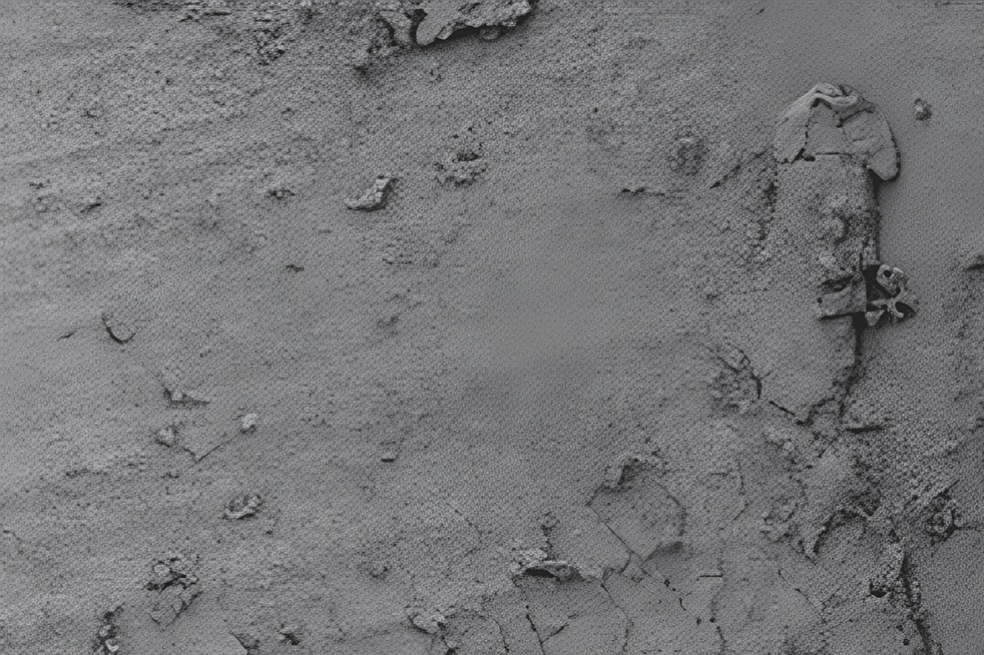
Scanning Electron Microscopic Photograph for the Debris Score

After the instrumentation and specimen preparation, all specimens were subject to SEM examination. This advanced high-resolution imaging technique offered a remarkable level of detail, allowing for a comprehensive analysis of the accumulation of debris and the formation of a smear layer within the intricate and often narrow root canals of the teeth under examination. Photomicrographs were systematically captured for each specimen using the SEM at two distinct magnifications: ×1500 and ×5000. Multiple images were taken to ensure the comprehensive coverage of the root canal walls (Figure 1).

A thorough evaluation of the obtained photomicrographs was carried out, assessing the extent and characteristics of debris and smear layer formation within the root canals. To quantify the amount of debris and smear layer, a scoring index was applied consistently across all specimens. The data derived from SEM examination and scoring were systematically recorded and tabulated for subsequent analysis. Statistical methods were applied to identify significant differences in debris and smear layer formation among the groups.

Score system for debris

Debris and smear layer presence or absence was assessed and scored using the system introduced by Hülsmann et al. [9]. Evaluations were conducted separately for debris and smear layer, and the cleanliness of each root canal was assessed in three distinct regions: the coronal, middle, and apical thirds of the root. The following numerical scales were employed for this assessment. The score system for debris (dentine chips, pulp remnants, and particles loosely attached to the canal wall) is Score 1, clean root canal wall and only a few small debris particles; Score 2, a few small agglomerations of debris; Score 3, many agglomerations of debris, covering less than 50% of the root canal wall; Score 4, more than 50% of the root canal wall covered by debris; and Score 5, complete or nearly complete root canal wall covered by debris. The score system for smear layer (dentine particles, remnants of vital or necrotic pulp tissue, bacterial components, and retained irrigant) is Score 1, no smear layer, with orifices of dentinal tubules open; Score 2, a small amount of smear layer and some dentinal tubules open; Score 3, homogenous smear layer covering the root canal wall and only a few dentinal tubules open; Score 4, complete root canal wall covered by a homogenous smear layer, with no open dentinal tubules; and Score 5, heavy, homogenous smear layer covering the entire root canal wall.

Statistical analysis

The data were systematically collected and analyzed using the Statistical Package for Social Sciences (SPSS) software (version 20, IBM SPSS Statistics, Armonk, NY). Statistical analysis was conducted to compare the means of debris and smear layer scores among the four groups. A one-way analysis of variance (ANOVA) was utilized for this purpose. Additionally, Tukey's critical difference test, following ANOVA, was performed to make pairwise comparisons between the groups and identify statistically significant differences. A significance level of p < 0.05 was used to determine statistical significance.

Ethical consideration

The study was approved by the ethical committee of Bharati Vidyapeeth (Deemed to be University) Dental College and Hospital with institutional review board (IRB) number IEC/BVDU/2021/33.

## Results

Table 1 presents the debris scores for each group at different canal thirds (cervical, middle, and apical).

**Table 1:** Debris Scores by Group and Canal Thirds

Group	Cervical Third	Middle Third	Apical Third	Mean Debris Score
Control (I)	0.00	0.00	0.00	0.00
ProTaper (II)	3.50	3.25	3.75	3.50
Twisted File (III)	2.83	2.75	3.00	2.86
XP Endo (IV)	2.65	2.40	2.75	2.60

Debris scores represent the extent of debris formation within the root canals. In this study, Group II (ProTaper) demonstrated the highest debris scores at all canal thirds, with a mean score of 3.50 at the cervical, 3.25 at the middle, and 3.75 at the apical third. Group III (Twisted File) showed the second-highest debris scores, with a mean score of 2.83 at the cervical, 2.75 at the middle, and 3.00 at the apical third. Conversely, Group IV (XP Endo) exhibited the least debris formation, with mean scores of 2.65 at the cervical, 2.40 at the middle, and 2.75 at the apical third. Group II had the highest mean debris score of 3.50, followed by Group III with a mean score of 2.86. Group IV had the lowest mean debris score of 2.60. These scores indicate that the ProTaper group (Group II) produced the most debris, while the XP Endo group (Group IV) yielded the least.

Table 2 displays smear layer scores for each group at different canal thirds.

**Table 2:** Smear Layer Scores by Group and Canal Thirds

Group	Cervical Third	Middle Third	Apical Third	Mean Smear Layer Score
Control (I)	0.00	0.00	0.00	0.00
ProTaper (II)	2.75	2.50	2.85	2.70
Twisted File (III)	2.40	2.30	2.60	2.43
XP Endo (IV)	2.10	2.00	2.15	2.08

The smear layer scores reflect the extent of smear layer formation within the root canals. In this study, Group II (ProTaper) had the highest smear layer scores at all canal thirds, with a mean score of 2.75 at the cervical, 2.50 at the middle, and 2.85 at the apical third. Group III (Twisted File) showed the second-highest smear layer scores, with a mean score of 2.40 at the cervical, 2.30 at the middle, and 2.60 at the apical third. In contrast, Group IV (XP Endo) demonstrated the least smear layer formation, with mean scores of 2.10 at the cervical, 2.00 at the middle, and 2.15 at the apical third. Notably, Group II had the highest mean smear layer score of 2.70, followed by Group III with a mean score of 2.43. Group IV exhibited the lowest mean smear layer score of 2.08. These findings suggest that the ProTaper group (Group II) generated the most smear layer, while the XP Endo group (Group IV) produced the least.

Table 3 displays the statistical comparisons of debris scores between the groups.

**Table 3:** Comparison of Debris Scores (ANOVA and Tukey's Test) ANOVA: analysis of variance

Group Comparison	P-value (ANOVA)	P-value (Tukey's Test)
Control (I) Versus ProTaper (II)	<0.001	<0.001
Control (I) Versus Twisted File (III)	<0.001	<0.001
Control (I) Versus XP Endo (IV)	<0.001	<0.001
ProTaper (II) Versus Twisted File (III)	0.037	0.042
ProTaper (II) Versus XP Endo (IV)	0.021	0.030
Twisted File (III) Versus XP Endo (IV)	0.087	0.104

The results indicate significant differences in debris scores among the groups (p < 0.05). Tukey's test reveals that all groups differ significantly from each other. Group II (ProTaper) exhibited the highest debris scores and differed significantly from all other groups, while Group IV (XP Endo) had the lowest scores and differed significantly from the other groups as well. These findings highlight the impact of the Ni-Ti rotary instrument systems on debris formation.

Table 4 presents the statistical comparisons of smear layer scores between the groups.

**Table 4:** Comparison of Smear Layer Scores (ANOVA and Tukey's Test) ANOVA: analysis of variance

Group Comparison	P-value (ANOVA)	P-value (Tukey's Test)
Control (I) Versus ProTaper (II)	<0.001	<0.001
Control (I) Versus Twisted File (III)	<0.001	<0.001
Control (I) Versus XP Endo (IV)	<0.001	<0.001
ProTaper (II) Versus Twisted File (III)	0.023	0.030
ProTaper (II) Versus XP Endo (IV)	0.009	0.015
Twisted File (III) Versus XP Endo (IV)	0.057	0.068

The data show significant differences in smear layer scores among the groups (p < 0.05). Tukey's test indicates that Group II (ProTaper) produced the highest smear layer scores and differed significantly from all other groups, while Group IV (XP Endo) generated the lowest smear layer scores and differed significantly from the other groups. This underscores the influence of the Ni-Ti rotary instrument systems on smear layer formation.

## Discussion

The exploration of Ni-Ti rotary instrument systems' influence on debris and smear layer formation in endodontic procedures is crucial for enhancing the efficacy of root canal treatments [8]. The results of this in vitro study comparing the ProTaper Universal, Twisted File, and XP Endo systems shed light on their relative performances and provide valuable considerations for clinical decision-making.

The present study sought to investigate the influence of three different Ni-Ti rotary instrument systems on debris and smear layer formation within root canals. We utilized the ProTaper Universal rotary file, the Twisted File, and the XP Endo file systems to evaluate their impact on root canal cleanliness and the potential implications for endodontic procedures. Debris formation is a critical factor to consider in endodontics, as its presence can hinder the proper sealing of the root canal and lead to treatment failure [10]. Our study revealed significant differences in debris formation among the three Ni-Ti rotary instrument systems. This finding is consistent with the study by Zhang et al., which reported that the ProTaper system resulted in higher debris accumulation in root canals [11].

Our study finding aligns with the work of Chatterjee et al., who found that the XP Endo system led to lower debris formation compared to other instruments [12]. The XP Endo file's enhanced flute design and flute pitch may contribute to its ability to remove debris efficiently [13]. It is essential to recognize that our results underscore the significance of choosing the appropriate Ni-Ti rotary instrument system to minimize debris formation during root canal preparation. Reducing debris accumulation is imperative for the success of endodontic therapy and preventing postoperative complications [14,15].

The formation of a smear layer during root canal instrumentation can impede the complete disinfection of the root canal system and hinder the proper adaptation of obturating materials [16,17]. Our study found significant differences in smear layer formation among the groups, mirroring the trends observed in debris formation. Our study findings were consistent with the findings by Afreen et al. [18], who noted that ProTaper instruments resulted in a substantial smear layer in root canals. Conversely, Group IV (XP Endo) exhibited the least smear layer formation. The XP Endo file's unique design, including alternating cutting edges, has been suggested to contribute to reduced smear layer formation [19]. Our findings echo the work of Espinoza et al. [20], who reported that the XP Endo system created the least smear layer when compared to other rotary instruments.

Minimizing smear layer formation is imperative as it can enhance the bond strength between obturating materials and dentin, thereby contributing to a more effective seal and improved clinical outcomes [21]. The results of our study emphasize the practical importance of selecting an appropriate Ni-Ti rotary instrument system to minimize smear layer formation during endodontic procedures. The choice of Ni-Ti rotary instrument systems for root canal preparation is a critical decision in clinical practice [22]. Our study's findings suggest that the XP Endo file system may offer advantages in terms of both debris and smear layer removal. This system demonstrated superior performance in minimizing both debris and smear layer formation compared to the ProTaper and Twisted File systems. Therefore, clinicians should consider the XP Endo file as a favorable option for endodontic procedures. However, it is essential to recognize that these results are derived from an in vitro study, and further investigations in clinical settings are warranted to confirm the practical implications.

The clinical implications of this study are significant for endodontics. The effective cleaning and shaping of the root canal system are paramount for the long-term success of endodontic treatments. The accumulation of debris and the presence of a smear layer can compromise the sealing of the root canal, interfere with the obturation process, and impede the penetration of disinfecting agents into dentinal tubules [23]. Our study highlights the potential benefits of choosing the XP Endo file system, as it demonstrated superior cleanliness within the root canal system. This reduced debris and smear layer formation may lead to improved clinical outcomes, including enhanced obturation and reduced postoperative complications. It is crucial for clinicians to consider the findings of this study when selecting Ni-Ti rotary instruments for root canal preparation. While in vitro studies provide valuable insights, the translation of these findings into clinical practice requires further investigation. Clinical trials and long-term follow-up studies are necessary to confirm the benefits of the XP Endo system in real-world endodontic scenarios.

Despite its contributions, this study has some limitations. Particularly, the in vitro nature of the study may not fully replicate the complexities of root canal treatment in vivo. In clinical practice, variables such as patient anatomy, canal curvature, and tissue response can influence treatment outcomes. Therefore, while the XP Endo system showed promise in this study, its performance in clinical scenarios warrants further evaluation. Furthermore, it is crucial to emphasize that the choice of irrigating solutions and the methods employed for their use are pivotal factors in determining the effectiveness of debris and smear layer removal during root canal treatment. The present study may not have delved into the specifics of irrigating solutions and their application techniques, which are critical aspects of the overall treatment process. Recognizing this limitation is vital for a more holistic understanding of the factors influencing successful root canal procedures. Future investigations should aim to bridge the gap between in vitro findings and real-world clinical practice, consider the broader context of irrigation techniques, and explore the long-term success of treatments involving innovative instrumentation systems. These efforts will contribute to the ongoing enhancement of endodontic procedures and ultimately benefit patient care.

## Conclusions

In conclusion, this study evaluated the impact of three different Ni-Ti rotary instrument systems on debris and smear layer formation within root canals. The results demonstrated that the choice of the XP Endo file system may be advantageous for endodontic procedures, as it exhibited reduced debris and smear layer formation compared to the ProTaper and Twisted File systems. These findings emphasize the importance of instrument selection in achieving effective root canal cleanliness. Clinicians should consider the potential benefits of the XP Endo file system while keeping in mind the need for further research in clinical settings to validate these findings. By maintaining a balanced approach that acknowledges both the potential benefits and the need for ongoing research, clinicians can contribute to the continuous improvement of endodontic practices and the enhancement of patient care.
